# In Search of a Function for BCLAF1

**DOI:** 10.1100/tsw.2010.132

**Published:** 2010-07-20

**Authors:** Haya Sarras, Solmaz Alizadeh Azami, J. Peter McPherson

**Affiliations:** Department of Pharmacology and Toxicology, University of Toronto, Canada

**Keywords:** BCLAF1, apoptosis, transcription, RS domain, lung development, Kaposi’s sarcoma, ribonucleoprotein, TRAP150, RNA transport

## Abstract

BCLAF1 was originally identified as a protein that interacts with antiapoptotic members of the Bcl2 family. Initial studies indicated a role for this protein as an inducer of apoptosis and repressor of transcription. Subsequent studies have shown that BCLAF1 plays criticals roles in a wide range of processes that are not normally associated with actions of Bcl2 family members, including lung development, T-cell activation, and control of the lytic infection program of Kaposi's sarcoma–associated herpesvirus. Here, we provide an overview of findings from past studies that both support and challenge the role of BCLAF1 in cell death and transcriptional control. We also present recent findings from our laboratory and others indicating a role for BCLAF1 in post-transcriptional processes that impact mRNA metabolism, instead of a direct role for this protein in apoptosis or transcription.

## BCLAF1 DISCOVERY AS AN E1B 19K INTERACTING PROTEIN, PROAPOPTOTIC FACTOR, AND TRANSCRIPTIONAL REPRESSOR

Our interest in BCLAF1 stems from initial studies by White and colleagues, who identified BCLAF1 (Bcl2 associated transcription factor; also known as BTF) in a screen for proteins that interact with the adenoviral E1B 19K protein[1]. E1B 19K is functionally similar to antiapoptotic Bcl2 members and blocks death signals triggered by other adenoviral proteins, such as E1A (reviewed in [2]). Although an interaction of BCLAF1 with E1B 19K and antiapoptotic BCL2 and BCL-xL was demonstrated *in vitro*, an interaction between BCLAF1 and Bcl2 family members could not be confirmed in cells using conventional molecular approaches as BCLAF1 overexpression caused apoptosis[1]. The ability of BCLAF1 to trigger apoptosis was blocked by coexpression of E1B 19K. In addition, BCLAF1 overexpression was found to suppress transcription from a heterologous reporter, an activity that could be suppressed in the presence of overexpressed E1B 19K, BCL2, or BCL-xL. Furthermore, the suppression of E1A-mediated transformation by BCLAF1, and the reduction or absence of BCLAF1 in tumor cell lines tested, suggested a potential role for this protein as a tumor suppressor[1]. It is not entirely clear whether the apoptotic and transcriptional activities of BCLAF1 that are blocked by BCL2 family members are indeed due to physical interaction between BCLAF1 and BCL2 proteins in a common subcellular compartment. Although E1B 19K is localized to the nuclear envelope, most BCL2 family members are localized to mitochondria, in contrast to the nuclear localization of BCLAF1[3].

Subsequent studies have also implicated BCLAF1 in pathways that link transcriptional events to cell death. BCLAF1 was identified in a screen for proteins that interact with emerin, a type-II inner nuclear membrane protein[4]. Emerin is encoded by the EMD gene located on the human X-chromosome, which, when mutated, gives rise to the X-linked form of Emery-Dreifuss muscular dystrophy[5]. Emerin is a lamin A/C binding protein that participates in nuclear envelope mechanics that impact chromosome segregation, gene expression, and muscle differentiation[6,7,8,9]. Although BCLAF1 and emerin were shown to interact in various *in vitro* assays, both proteins were found to reside in distinct subcellular compartments. BCLAF1 was observed to be concentrated in focal subnuclear dots or speckles, unlike emerin, which localized to the nuclear envelope as expected. Following induction of apoptosis, some of the BCLAF1 was observed to redistribute to the nuclear envelope with emerin. It is presently unclear whether this colocalization reflects a causative role for these proteins in apoptosis or is simply a consequence of cell death. BCLAF1 has also been linked to transcription of p53 through a reported interaction with and activation by protein kinase Cδ, using chromatin immunoprecipitation assays[10]. Although the activity of this kinase has been shown to trigger apoptosis and cell cycle arrest in response to DNA damage[11], p53 activation is chiefly linked to post-translational control of p53 protein stability and conformation, as opposed to signaling that modifies transcriptional control of p53 expression (reviewed in [12,13]). A more recent study identified BCLAF1 in a screen for changes in steady-state protein levels following exposure of HCT116 cells to ceramide[14]. In this study, BCLAF1 protein levels were found to be induced approximately twofold following exposure to ceramide. BCLAF1 depletion by siRNA conferred resistance to ceramide-induced apoptosis, whereas overexpression of BCLAF1 was capable of inducing p53 and BAX levels, but down-regulating MDM2 levels. Taken together, these studies potentially support a role for BCLAF1 in apoptosis through events that control transcription. The exact function of BCLAF1 in these events, however, remains unclear.

## ROLES FOR Bclaf1 IN DEVELOPMENT INDEPENDENT OF APOPTOSIS

In order to gain insight into the importance of Bclaf1 *in vivo*, we generated Bclaf1-deficient mice[15]. Bclaf1 is dispensable for cell viability, but is essential for postnatal viability in mice. No embryonic lethality was observed in the absence of Bclaf1; however, we discovered that Bclaf1-deficient mice exhibit a striking arrest in lung development resulting in death shortly after birth. Lung formation as a developmental process can be considered as a series of stages that initiate on mouse embryonic day 9.5 (E9.5) and continue after birth (reviewed in [16,17]). Formation of the bronchiole tree occurs during the initial pseudoglandular stage (E9.5–E16.6) with subsequent branching of the bronchial buds occurring by E12. The canalicular stage (E16.6–E17.4) is characterized by extensive branching of the distal epithelium and mesenchyme, which leads to the formation of terminal sac structures at the tips of the bronchiolar tree. During the saccular stage (E17.5 to postnatal day 5), expansion of these terminal sacs is accompanied by a corresponding decrease in interstitial tissue. This stage is required for the formation of a functional blood-air barrier, and is characterized by differentiation of bronchoalveolar epithelial cells into type-I and type-II lineage cells that are critical for fluid removal and surfactant production, respectively. The final alveolar stage is initiated postnatally, and is characterized by maturation of alveoli and formation of secondary alveolar septa.

The lungs of mice deficient in Bclaf1 arrest during the saccular stage with an overabundance of mesenchyme and lack terminal sac expansion. We generated an antibody against Bclaf1 and discovered that expression of this protein in the lung was markedly up-regulated during the initiation of the saccular stage at E17.5. The saccular stage is a critical period for differentiation of epithelial lineage cells that line the terminal sacs. Bclaf1 deficiency did not impair differentiation of type-I and type-II epithelial cells, but interestingly resulted in an overabundance of smooth muscle cells throughout the lung. No significant difference in proliferation or apoptosis was observed when lungs from Bclaf1-deficient mice were compared to wild-type siblings, suggesting that the role of Bclaf1 in lung differentiation is not linked to a general effect on cell growth or cell death. Interestingly, Bclaf1-deficient neonates also tended to exhibit polydactyly and T cells were found to have an activation-dependent proliferation defect *ex vivo*. Bclaf1 was found to play critical roles in development and cell homeostasis, but, importantly, these roles could not be linked to a role for Bclaf1 in apoptosis.

To gain further insights into the role of Bclaf1 in lung development, we compared global mRNA expression patterns of Bclaf1-deficient vs. wild-type lungs at postnatal day 1. We conducted microarray hybridization experiments using postnatal day 1 lung mRNA obtained from six sibling pairs of wild-type vs. *Bclaf1*$^{-/-}$ mice. We discovered that compared to wild-type lungs, 1087 annotated transcripts were up-regulated, whereas 1229 were down-regulated in Bclaf1-deficient lungs. By statistical analysis of gene ontology (GO) and Kyoto Encyclopedia of Genes and Genomes (KEGG) pathway terms assigned to genes that demonstrated significant changes in expression, we identified several pathways and processes with altered expression in Bclaf1-deficient lungs[18] (Table 1). We found the following GO gene annotations and KEGG pathway terms to be over-represented: muscle contraction, uteric bud (kidney) formation, and Wnt signaling. Interestingly, GO annotations corresponding specifically to lung development or apoptosis were not over-represented. Although Bclaf1 was initially characterized as a transcriptional repressor and an inducer of apoptosis, our profiling analysis did not reveal any notable change in gene expression that could signify or substantiate a role for Bclaf1 in transcriptional repression of genes linked to apoptosis, as has been suggested in previous studies. Surprisingly, 121 of the genes represented in our microarray experiments by multiple expressed sequence tag (EST) clones were found to be significantly up-regulated for some EST clones, whereas other EST clones for the same gene were significantly down-regulated. Closer inspection of these ESTs revealed they mapped to different regions of the mRNAs that have previously been demonstrated to undergo alternative splicing or changes in 3' end formation. Our laboratory is currently investigating whether these changes in abundance of transcript isoforms may reflect a role for Bclaf1 in processes that govern pre-mRNA splicing and/or processing that are critical for development.

## STRUCTURAL FEATURES AND SUBCELLULAR LOCALIZATION OF BCLAF1

Although linked to (and named for its) interaction with BCL2 family members, BCLAF1 does not share structural similarities with these proteins[1]. The most prominent feature of the BCLAF1 open reading frame is the presence of an arginine-serine rich (RS) region located near the N-terminus. RS domain–containing proteins are typically linked with pre-mRNA biogenesis and processing events, such as pre-mRNA splicing. For example, SR proteins are a large class of factors with RS domains that play pivotal roles in the control of pre-mRNA splicing and mRNA processing events. SR proteins typically contain N-terminal RNA recognition motifs that bind pre-mRNA and C-terminal RS domains, which mediate interactions with other RS domain–containing proteins[19,20,21].

Furthermore, the subcellular localization of BCLAF1 does not coincide with the localization of its reported protein partners. The first report to describe subcellular localization of endogenous BCLAF1 revealed its nuclear organization to be focal, reminiscent of the nuclear "speckle" pattern demonstrated by various proteins linked to pre-mRNA splicing and processing events that accumulate in complexes known as interchromatin granule clusters[4,22,23,24,25]. Despite these similarities in structure and subcellular localization to pre-mRNA splicing factors, a role for BCLAF1 in regulating pre-mRNA splicing or mRNA processing events has not previously been examined.

## BCLAF1 AS A COMPONENT OF RIBONUCLEOPROTEIN (RNP) COMPLEXES

Eukaryotic conversion of pre-mRNA into mature mRNA requires the careful coordination of various post-transcriptional events, such as pre-mRNA 5' capping, splicing, polyadenylation, and mRNA export from the nucleus to the cytoplasm. The stepwise completion of each phase is orchestrated by a dedicated repertoire of molecular factors that are sequentially recruited to the RNA substrate. These factors interact

## TABLE 1
## GO and KEGG Term Analysis*

**GO term: muscle contraction:** 17 genes either significantly up- or down-regulated (EASE score = 1.2e-3), with 12 of these 17 genes significantly up-regulated (EASE score = 2.2e-3)

| ***Up-regulated*** | | ***Down-regulated*** | |
|---|---|---|---|
| *Adrb2* | adrenergic receptor, beta 2 | *Myh6* | myosin, heavy chain 6 |
| *Anxa6* | annexin A6 | *Prkca* | protein kinase c, alpha |
| *Arhgef11* | rho guanine exchange factor 11 | *Tpm1* | tropomyosin 1 |
| *Atp1a2* | Na+/K+ atpase, alpha 2 | *Tpm2* | tropomyosin 2 |
| Atp2a2 | Ca2+ atpase, slow twitch 2 | *Utrn* | utrophin |
| *Capn3* | calpain 3 | | |
| *Casq1* | calsequestrin 1 | | |
| *Casq2* | calsequestrin 2 | | |
| *Cald* | caldesmon | | |
| *Myh11* | myosin heavy chain 11 | | |
| *Tnnt2* | troponin T2, cardiac | | |
| *Tpm3* | tropomyosin 5 | | |

**GO term: uteric bud (kidney) development:** 7 genes down-regulated (EASE score = 2.3e-3)

| ***Down-regulated*** | |
|---|---|
| *Agt* | angiotensinogen |
| *Bmp2* | bone morphogenetic protein 2 |
| *Gpc3* | glypican 3 |
| *Pbx1* | pre b-cell leukemia transcription factor 1 |
| *Rara* | retinoic acid receptor, alpha |
| *Rarb* | retinoic acid receptor, beta |
| *Robo2* | roundabout homolog 2 (*Drosophila*) |

**KEGG pathway term: Wnt signaling:** 13 genes up-regulated and 9 genes down-regulated (EASE score = 3.5e-2). *NOTE:* for Crebbp, two ESTs up-regulated, one EST down-regulated.

| ***Up-regulated*** | | ***Down-regulated*** | |
|---|---|---|---|
| *Camk2d* | protein kinase, camp dependent | *Btrc* | beta-transducin repeat cont. |
| *Crebbp* | creb binding protein | *Ccnd1* | cyclin D1 |
| *Csnk1a1* | casein kinase, alpha 1 | *Crebbp* | creb binding protein |
| *Csnk2a1* | casein kinase II, alpha 1 | *Lef* | lymphoid enhancer factor 1 |
| *Csnk2a2* | casein kinase II, alpha 2 | *Lrp6* | LDL receptor-related 6 |
| *Csnk2b* | casein kinase II, beta subunit | *Prkca* | protein kinase c, alpha |
| *Ctnnb1* | catenin, beta 1 | *Psen1* | presenilin 1 |
| *Cul1* | cullin 1 | Tcf7l2 | transcription factor 7-like 2 |
| *Daam1* | disheveled associated | *Wnt4* | wingless-related 4 |
| *Fbxw11* | f-box and wd-40 domain protein 11 | | |
| *Rhoa* | ras homolog gene family, member a | | |
| *Tcf3* | transcription factor 3 | | |
| *Tcf4* | transcription factor 4 | | |

* Individual annotated transcripts listed in each category showed a significant fold-change in expression calculated using LimmaGUI software[66]. GO analysis was performed using Database for Annotation, Visualization and Integrated Discovery (DAVID) tools (http://david.abcc.ncifcrf.gov/). Fisher's exact test–based *p* values (EASE score) were used to indicate the significance of the association with gene ontology terms. A cutoff of <0.05 was considered statistically significant. Data are available in Gene Expression Omnibus (GEO) as platform GPL5784, Samples GSM229428 and GSM225694, series GSE9034 (http://www.ncbi.nlm.nih.gov/geo/).

with RNA in RNP complexes that are initially formed during transcription. The protein composition of RNPs is remodeled during each phase of its existence in a dynamic fashion in order to dictate the fate of the contained RNA molecule (reviewed in [26]).

A recent study that examined the composition of human mRNPs using LC-MS/MS discovered the presence of BCLAF1 among newly identified mRNP proteins[27]. The association of BCLAF1 with mRNPs occurred independently of splicing, but was found to be dependent on the presence of CBP80/CBP20, proteins that form the 5'-m7G cap binding complex. The m7G cap is one of the earliest modifications to nascent transcripts and has been demonstrated to play pivotal roles in several stages of mRNA metabolism, including pre-mRNA splicing, 3' end formation and RNA transport, translation, and nonsense-mediated decay[28,29].

A subsequent study identified BCLAF1 in a complex that mediated cyclin D1 message stability together with SNIP1, SkIP, TAP150, and Pinin[30]. SNIP1 was found to be required for the recruitment of the RNA processing factor U2AF65 to cyclin D1 transcripts. The expression of SNIP1 together with associated proteins was observed to fluctuate as a function of cell cycle position, with levels highest in G1, but decreasing during S and G2 phases, which coincided with the timing of increased cyclin D1 mRNA accumulation. Taken together, these studies implicate BCLAF1 as a participant in processes that govern RNA metabolism; however, the precise role of BCLAF1 in these events remains unclear.

Studies from our laboratory support a role for Bclaf1 in RNA metabolism. We found that Bclaf1 coprecipitates with hnRNP A1, an RNP protein that is bound to cellular RNAs from transcription to translation (Fig. 1). RNPs can be immunoprecipitated using anti-hnRNP A1 from different subcellular fractions (nuclear pellet, nuclear soluble, cytoplasmic) that are associated with different stages of RNA metabolism. RNPs in the nuclear pellet are chromatin-associated and contain both pre-mRNA and mRNA, whereas RNPs from the nuclear soluble and cytoplasmic fractions contain predominantly mRNAs[31]. Immunoprecipitated Bclaf1 protein was found to be associated with hnRNP A1–containing complexes from the nuclear pellet, but not the soluble nuclear and cytoplasmic fractions, suggesting that Bclaf1 associates with RNPs containing pre-RNA, but not RNPs containing transcripts at later stages of maturation (Fig. 1).

Bclaf1 is unlikely to be essential for pre-mRNA splicing or mRNA processing, but could conceivably serve a regulatory or accessory role as *Bclaf1*$^{-/-}$ mice do not display early embryonic lethality as might be expected for a factor essential for RNA metabolism. Using a prototypical substrate for measuring alternative pre-mRNA splicing, we discovered that Bclaf1-deficient fibroblasts have altered levels of spliced transcripts derived from adenovirus E1A pre-mRNA compared to wild-type cells. Alternative splicing of E1A pre-mRNA generates three major species (13*S*, 12*S*, and 9*S*) through the use of three alternative 5' splice sites, in addition to two minor species (10*S* and 11*S*)[32,33,34,35,36]. In order to ascertain whether Bclaf1 deficiency is sufficient to impact splicing profiles, we compared the relative abundance of E1A mRNA species generated in wild-type and *Bclaf1*$^{-/-}$ fibroblasts (Fig. 2). When compared to wild-type cells, *Bclaf1*$^{-/-}$ cells showed increased relative levels of 12*S*, 10*S*, and 9*S* E1A RNAs, equivalent levels of 13*S* and 11*S*, and corresponding decreased levels of pre-mRNA (Fig. 2). These findings suggest that Bclaf1 may participate in pathways that negatively regulate alternative splicing and that aberrations in such pathways could be responsible for the pleiotrophic defects observed in Bclaf1-deficient mice. It is tempting to speculate a role for Bclaf1 in regulating alternative splicing events that could explain its role in lung development and other processes. Nevertheless, further studies measuring the ability of Bclaf1 to complement splicing-deficient protein extracts or modify alternative splice site selection *in vitro* will be required in order to determine a direct role conclusively for Bclaf1 in regulating pre-mRNA splice site selection.

While screening for other RNP components that interacted with BCLAF1, we observed colocalization and interaction with the RNA export factor TAP/NXF1. Indirect immunofluorescence of cells using antibodies against BCLAF1 and TAP1 revealed that both proteins exist in superimposable focal subcellular regions (Fig. 3). We also detected BCLAF1 in complexes from cells transfected with FLAG-TAP and immunoprecipitated using anti-FLAG (Fig. 3). TAP/NXF1 together with NXT1/p15 form a mRNP nuclear transport receptor that participates in the export of nuclear mRNP cargoes across nuclear pore complexes to the cytoplasm (reviewed in [37,38]). Further studies will be required to determine if BCLAF1 plays a direct role in mRNP export.

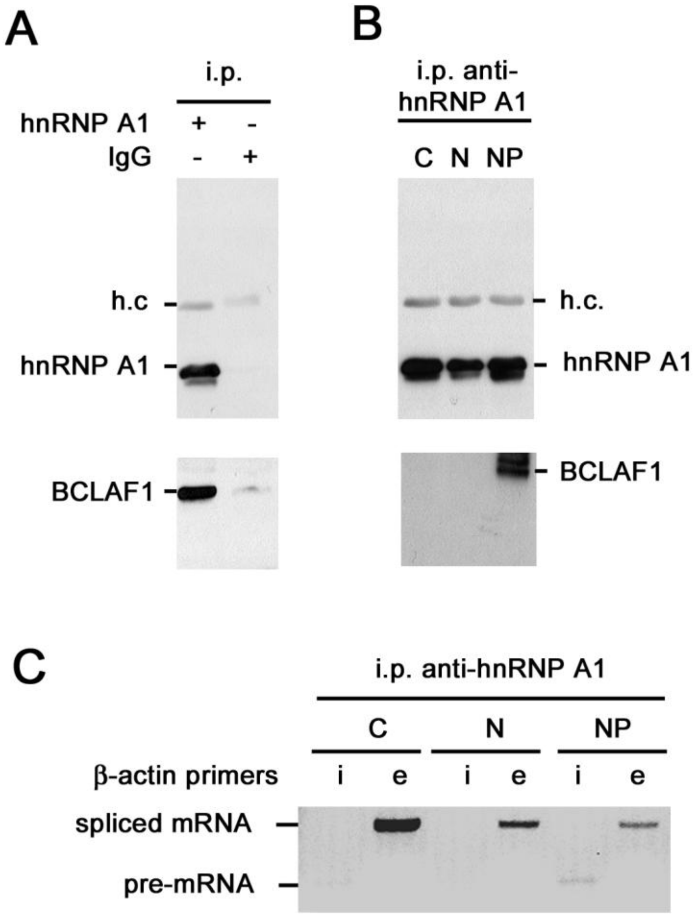

**FIGURE 1.** BCLAF1 associates with RNPs. (A) BCLAF1 coimmunoprecipitates with hnRNP A1. HeLa cell lysates subjected to immunoprecipitation with either anti-hnRNP A1 or control IgG (h.c. = heavy chain) were analyzed by Western analysis with anti-hnRNP A1 and anti-BCLAF1. (B) RNPs immunoprecipitated from cytoplasmic (C), nuclear soluble (N), and nuclear pellet (NP) fractions by anti-hnRNP A1 antibody (upper panel). BCLAF1 is contained with the fraction of RNPs in the nuclear pellet (NP, lower panel). (C) RT-PCR products of endogenous β-actin RNA from RNPs immunoprecipitated by anti-hnRNP A1 from fractions (C, N, and NP) validates that NP fractions of RNPs contain pre-mRNA (i) and spliced mRNAs (e).

## STRUCTURAL SIMILARITY OF BCLAF1 TO TRAP150

Our laboratory and others have noted that Bclaf1 shows a striking degree of protein sequence similarity to another recently described protein known as thyroid hormone receptor–associated protein 150 (TRAP150, also known as THRAP3). TRAP150 was originally identified in nuclear receptor transactivation complexes[39,40]. TRAP150 contains an RS domain like BCLAF1 and exhibits extensive sequence similarity with BCLAF1/Bclaf1, particularly in the C-terminal region of the protein, which is 48% identical to BCLAF1[24]. Interestingly, BCLAF1 and TRAP150 were found to both reside in interchromatin granule clusters[25], and affinity purification of RNP complexes demonstrated the presence of both BCLAF1 and TRAP150[27].

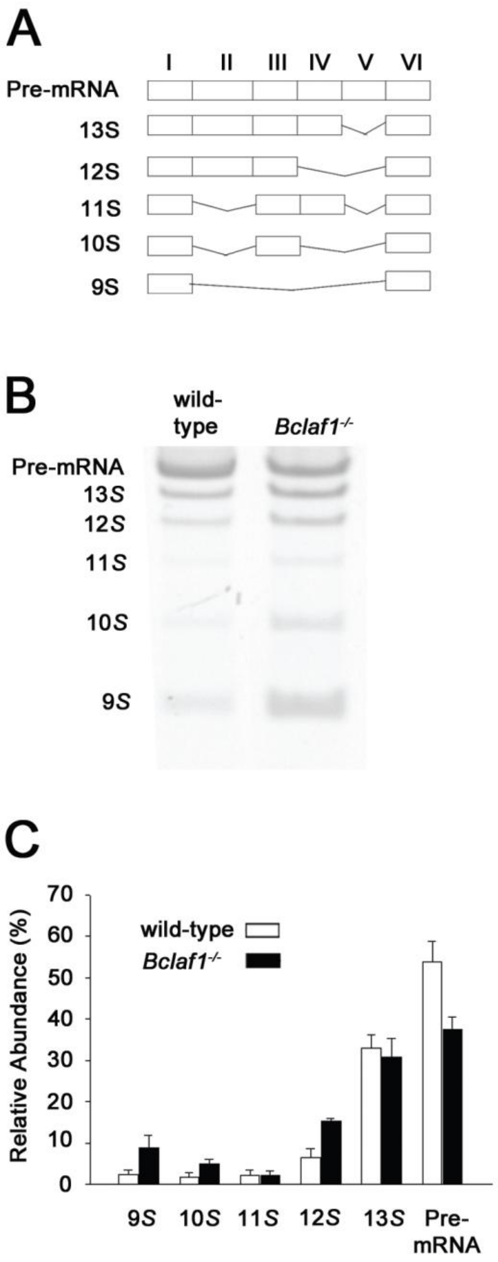

**FIGURE 2.** Bclaf1 deficiency impacts alternative splicing profiles. (A) Diagram of E1A pre-mRNA showing alternative splicing events that generate 13*S*, 12*S*, 11*S*, 10*S*, and 9*S* mRNAs. (B) Representative photomicrograph of TBE-PAGE gel showing spliced transcripts generated from embryonic fibroblasts (wild-type or *Bclaf1*$^{-/-}$) transfected with E1A minigene. (C) Relative levels of E1A mRNA species in wild-type (white bars) or *Bclaf1*$^{-/-}$ (black bars) embryonic fibroblasts. Error bars represent standard deviation of three independent experiments. Wild-type or *Bclaf1*$^{-/-}$ embryonic fibroblasts were transiently transfected with pCMVE1A[67], reverse-transcribed, and amplified by PCR over a range of 9 to 30 cycles. Reactions were electrophoresed in 5% TBE-PAGE gels and amplified reaction products were visualized following staining with Vistra-Green (GE Healthcare). Imaging of reaction products was captured using a PhosphorImager and quantified using ImageQuant software (Molecular Dynamics). The log intensity of reaction products was plotted vs. cycle number, and quantification was performed on reaction products in the linear range of amplification.

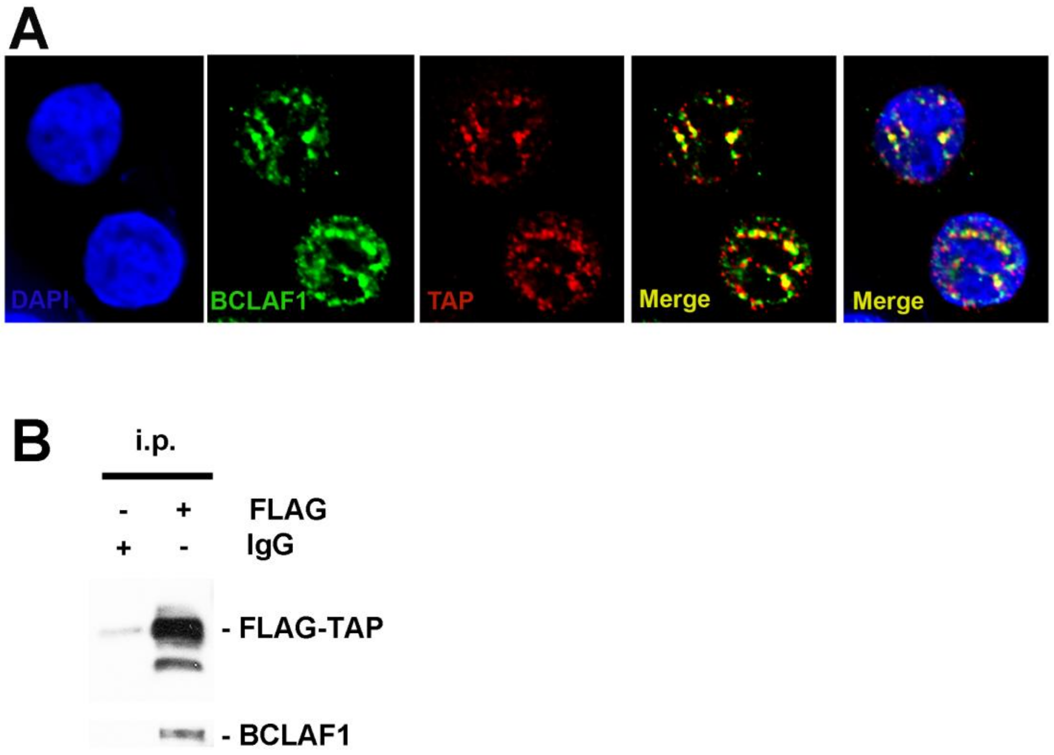

**FIGURE 3.** BCLAF1 interaction with TAP. (A) Indirect immunofluorescence staining of BCLAF1 and TAP. Representative micrograph showing DAPI-stained nucleus of HeLa cells (blue, DAPI) stained with anti-BCLAF1 (green, BCLAF1), and anti-TAP (red, TAP). Merged images show colocalization of endogenous BCLAF1 and TAP (Merge, yellow). (B) Coimmunoprecipitation of BCLAF1 with TAP. Protein lysate from HeLa cells transfected with FLAG-TAP was incubated with either control IgG or anti-FLAG. Western analysis of immunoprecipitated complexes was performed with anti-FLAG, followed by anti-BCLAF1.

TRAP150 was also recently identified in a complex with TAP/NXF1[41]. Although it is not clear whether TRAP150 participates in mRNA export pathways, increased production of TRAP150 in cells promoted pre-mRNA splicing of heterologous reporters, whereas depletion of this protein led to reduced splicing. Interestingly, the impact of splicing by TRAP150 in cells was not observed when TRAP150-depleted extracts were utilized in *in vitro* splicing assays. TRAP150 also facilitated mRNA degradation when tethered to a reporter mRNA; however, this activity occurs in the nucleus and is not linked to nonsense-mediated decay. The authors also demonstrated that overexpressed BCLAF1 could also promote pre-mRNA splicing and mRNA degradation in the nucleus. It will be interesting to determine whether BCLAF1 and TRAP150 participate in identical or related pathways governing regulation of pre-mRNA splicing or mRNA stability.

## BCLAF1 AS A TARGET FOR KAPOSI'S SARCOMA–ASSOCIATED HERPESVIRUS microRNAS

Surprisingly, a recent study has shown BCLAF1 to be a cellular target for microRNAs (miRNAs) encoded by Kaposi's sarcoma–associated herpesvirus (KSHV). KSHV is the etiological agent responsible for certain acquired immunodeficiency syndrome–related malignancies, such as Kaposi's sarcoma, primary effusion lymphoma, and variants of multicentric Castleman disease[42,43,44,45,46]. The KSHV genome encodes a miRNA cluster that is expressed from a single locus during viral latency (reviewed in [47]). A number of these miRNAs have been found to reduce the expression of various target genes, such as thrombospondin[48], BACH1[49,50], the transcription factor MAF[51], and IκBα[52]. One of these miRNAs, known as miR-K5, was found to target a sequence in the 3'-untranslated region of *BCLAF1* and down-regulate BCLAF1 expression[53]. Further analysis revealed that BCLAF1 could be down-regulated by several other miRNAs. Interestingly, when lytic KSHV growth was induced, inhibition of miRNAs was associated with increased BCLAF1 expression and decreased production of KSHV virions. Furthermore, depletion of BCLAF1 increased spontaneous lytic reactivation of KSHV. Taken together, these findings indicate a negative regulatory role for BCLAF1 in lytic viral replication.

The ability to hijack RNA export pathways is crucial for lytic replication of several viruses (reviewed in [54]). Several KSHV mRNAs expressed during the lytic phase of infection lack introns and are exported from the nucleus in a TAP/NXF1-dependent manner by a virus-encoded protein known as ORF57[55,56,57,58]. ORF57 has recently been shown to recruit the transcription/export (TREX) complex to intronless viral RNAs through a direct interaction between ORF57 and the TREX adaptor protein ALY/REF[59]. Given that BCLAF1 could have a role in mRNA processing events, such as RNA transport, the ability of BCLAF1 to thwart lytic viral replication might be through a mechanism that impedes ORF57-linked RNA transport during lytic viral replication.

## CHALLENGES FOR FUTURE RESEARCH

BCLAF1 has been reported to play a critical role in a variety of seemingly unrelated processes that include apoptosis, transcriptional control, pulmonary smooth muscle development, T-cell activation–dependent proliferation, and KSHV lytic viral replication. Our ability to move forward in understanding the role of Bclaf1 in these processes requires a careful re-evaluation of its proposed primary role as a proapoptotic factor that is controlled by Bcl2 family members. Recent progress suggests a potential regulatory role for this protein in processes that govern mRNA processing events – a compelling concept given that pre-mRNA splicing and processing are known to play a pivotal role in the regulation of apoptosis[60], the mRNA expression profile of smooth muscle[61,62,63,64], T-cell homeostasis[65], and factors that govern viral replication events[55,56,57,58]. A mechanistic understanding of BCLAF1 and the pathway in which it acts will provide valuable insight into molecular circuitry that impacts human health and disease.

## ACKNOWLEDGMENTS

We thank B. Chabot for pCMVE1A and J. Steitz for FLAG-TAP vectors. This work was supported by the Ontario Thoracic Society/Canadian Lung Association and the Province of Ontario Early Researcher Award to J.P.M. J.P.M. is a Canadian Insititutes for Health Research New Investigator.
